# Genetic Variants in One-Carbon Metabolism and Their Effects on DHA Biomarkers in Pregnant Women: A Post-Hoc Analysis

**DOI:** 10.3390/nu14183801

**Published:** 2022-09-15

**Authors:** Aura (Alex) P. Loinard-González, Olga V. Malysheva, Kevin C. Klatt, Marie A. Caudill

**Affiliations:** 1 Biological Sciences, Cornell University, Ithaca, NY 14853, USA; 2 Division of Nutritional Sciences, Cornell University, Ithaca, NY 14853, USA; 3 Nutritional Sciences & Toxicology, University of California, Berkeley, CA 94720, USA

**Keywords:** choline, DHA, pregnancy, genetic variants, one-carbon metabolism, PEMT

## Abstract

The delivery of docosahexanoic acid (DHA) to the fetus is dependent on maternal one-carbon metabolism, as the latter supports the hepatic synthesis and export of a DHA-enriched phosphatidylcholine molecule via the phosphatidylethanolamine *N*-methyltransferase (PEMT) pathway. The following is a post-hoc analysis of a choline intervention study that sought to investigate whether common variants in one-carbon metabolizing genes associate with maternal and/or fetal blood biomarkers of DHA status. Pregnant women entering their second trimester were randomized to consume, until delivery, either 25 (*n* = 15) or 550 (*n* = 15) mg choline/d, and the effects of genetic variants in the *PEMT*, *BHMT*, *MTHFD1*, and *MTHFR* genes on DHA status were examined. Variant (vs. non-variant) maternal *PEMT* rs4646343 genotypes tended to have lower maternal RBC DHA (% total fatty acids) throughout gestation (6.9% vs. 7.4%; main effect, *p* = 0.08) and lower cord RBC DHA at delivery (7.6% vs. 8.4%; main effect, *p* = 0.09). Conversely, variant (vs. non-variant) maternal *MTHFD1* rs2235226 genotypes exhibited higher cord RBC DHA (8.3% vs. 7.3%; main effect, *p* = 0.0003) and higher cord plasma DHA (55 vs. 41 μg/mL; main effect, *p* = 0.05). Genotype tended to interact with maternal choline intake (*p* < 0.1) to influence newborn DHA status for *PEMT* rs4646343 and *PEMT* rs7946. These data support the need to consider variants in one-carbon metabolic genes in studies assessing DHA status and requirements during pregnancy.

## 1. Introduction

Docosahexanoic acid (DHA) is a long-chain, omega 3 polyunsaturated fatty acid (22:6n-3) necessary for normal brain growth and cognitive development [1,2]. Although data are mixed [3], DHA supplementation is recommended throughout pregnancy to support fetal cognitive and retinal development [4] with emerging evidence supporting a protective effect on birth outcomes, such as preterm birth [5].

DHA circulates in several forms with a significant fraction found esterified within phosphatidylcholine (PC) molecules; such DHA-enriched PCs are a major end-product of the hepatic phosphatidylethanolamine *N*-methyltransferase (PEMT) pathway [6]. The PEMT pathway is a major consumer of one-carbon (methyl) units derived from nutrients such as choline and folate. Indeed, supplementation with choline has been shown to improve DHA bioavailability in women of reproductive age [7] as well as pregnant women [8] by bolstering methyl group supply for PEMT activity.

**Figure 1:**
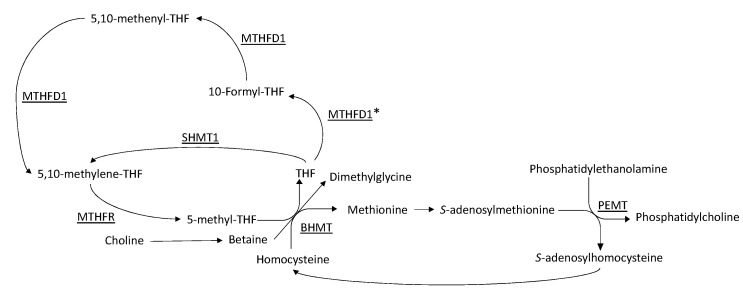
The role of one-carbon metabolism in phosphatidylcholine synthesis through the phosphatidylethanolamine *N*-methyltransferase (PEMT) pathway. Key enzymes are underlined. BHMT: betaine-homocysteine *S*-methyltransferase; MTHFD1: methylenetetrahydrofolate dehydrogenase; MTHFR: methylenetetrahydrofolate reductase; PEMT: phosphatidylethanolamine *N*-methyltransferase; SHMT1: serine hydroxymethyltransferase 1. * Denotes the domain of the MTHFD1 tri-functional enzyme containing the rs2235226 variant.

The presence of common variants in genes involved in the metabolism of choline and folate have the potential to influence the bioavailability of DHA. For example, genes, such as *BHMT*, *MTHFD1*, and *MTHFR*, all play a central role in the provision of methyl groups ultimately utilized by the PEMT pathway (Figure 1). Notably, variants within these genes, as well as common variants within *PEMT*, have previously been shown to influence biomarkers of both choline and folate metabolism [9,10,11]. Thus, the current analysis sought to investigate whether common variants in one-carbon metabolizing genes associate with maternal and/or fetal blood biomarkers of DHA status. The interactive effects of genotype and maternal choline intake were also assessed. Ultimately, these data, and future analyses spurred by this investigation, are expected to inform more individualized dietary recommendations and contribute to the growing field of precision nutrition.

## 2. Materials and Methods

### 2.1. Study Design and Participants

The current study is a post-hoc analysis of a randomized choline intervention study [8]. Healthy pregnant women (21–40 years of age, BMI < 32, and between 12–16 gestational weeks) were recruited in Ithaca, NY, between October 2017 and April 2019. Participants were randomized into either a control group (25 mg choline/d; *n* = 15) or an intervention group (550 mg choline/d; *n* = 15) from gestational week 12–16 through delivery. Throughout the study, all participants consumed a self-selected diet along with a daily 200 mg DHA supplement (Nature’s Way EfaGold Neuromins 200 mg DHA [plant source]; DSM Nutritional Products; Heerlen, The Netherlands), and an over-the-counter prenatal vitamin/mineral supplement (Nature Made Prenatal Tablet; Pharmavite LLC; San Fernando, CA, USA). Compliance with the study protocol was monitored via the return of supplement containers. The study was approved by the Institutional Review Board for Human Study Participant Use at Cornell University in Ithaca, NY, USA and Cayuga Medical Center in Ithaca, NY, USA (where women delivered their babies). All participants gave written informed consent before enrollment.

### 2.2. Data Collection

Participants visited the Human Metabolic Research Unit (HMRU) at Cornell University (Ithaca, NY, USA) three times throughout their pregnancy. The first visit was between weeks 12 and 16; the second, between weeks 20 and 24; and the third between weeks 28 and 32. During each visit, participants were given their daily supplements (described above) and provided a fasting blood sample. Maternal blood and fetal cord blood were collected at delivery.

### 2.3. Genotyping and Analytical Measurements

DNA was extracted from blood buffy coat using a DNEasy kit (DNEasy Blood and Tissue Kit; Qiagen, Hilden, Germany), and re-precipitated with ethanol to increase quality and concentration. Five single nucleotide polymorphisms (SNPs), rs4646343 and rs7946 in the *PEMT* gene, rs11081133 in the *MTHFR* gene, rs2236225 in the *MTHFD1* gene, and rs3733890 in the *BHMT* gene, were determined using a fluorescence-based hybridization assay with a master mix (TaqMan^TM^ Genotyping Master Mix; catalog Number: 4371355; Thermofisher Scientific; Waltham, CA, USA) and probe (TaqMan^TM^ Genotyping Assay, human; catalog number 4351379; Thermofisher Scientific; CA, USA) according to the instructions of the manufacturer.

Fatty acid composition of maternal and cord washed RBCs, as well as maternal and cord plasma, was measured via gas chromatography (GC) coupled to a flame ionization detector at OmegaQuant^®^ [8]. DHA content is expressed as a percent of total fatty acids in RBCs, and as an absolute concentration in plasma.

### 2.4. Statistical Analysis

All statistical analyses were performed with R (version 1.1.419). Mixed linear models were employed to examine the effect of maternal genotype on maternal RBC and maternal plasma DHA content. Each model included genotype, choline intervention arm, and time (study visit) as fixed effects and participant ID as a random effect. Additionally, the interaction terms, genotype x time and genotype x choline intervention arm, were included to assess whether the effect of genotype was modified by either study time-point or maternal choline intake. Generalized linear models were utilized to assess the relationship of maternal and newborn genotype to cord RBC and cord plasma DHA content; fixed effects included genotype and choline intervention arm, and interactive effects between these two variables was considered. All *p*-values ≤ 0.05 were considered significant; given the exploratory nature of the study, *p*-values less than 0.15 were considered as trends.

Line plots were made using the least square means (LSMs) data, with confidence intervals as error bars, and box plots were made using raw data. Visualization was performed using the R ggplot2 package.

## 3. Results

### 3.1. Participant Characteristics

This post-hoc analysis included genotype data from 30 pregnant women and 28 of their newborns. The frequency of maternal and newborn genotypes in each choline intervention arm are shown in Table 1, while demographic and clinical characteristics by maternal genotype are shown in Table 2. The frequency of variants and non-variants did not differ (*p* > 0.05) by choline intervention arm, and no differences (*p* > 0.05) were detected in the demographic and clinical characteristics within a maternal genotype.

### 3.2. Study Outcomes

The outputs of the regression models for each genotype (maternal and newborn) are shown in Supplementary Table S1 (*p*-values for maternal genotype), Table S2 (*p*-values for newborn genotype), and Table S3 (DHA outcome values).

#### 3.2.1. *PEMT* rs4646343

**Figure 2:**
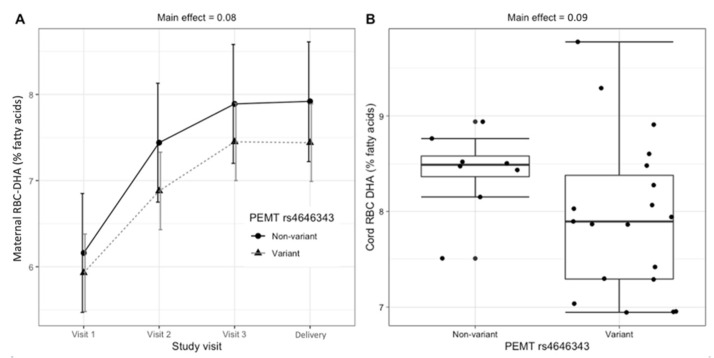
Relationship between maternal *PEMT* rs4646343 genotype and RBC-DHA (% of total fatty acids). (**A**) Maternal RBC-DHA throughout gestation (LSM ± confidence interval); (**B**) cord RBC-DHA at delivery (raw data boxplot).

Carriers (versus non-carriers) of the maternal *PEMT* rs4646343 variant exhibited borderline lower DHA as a percentage of total fatty acids in RBCs (RBC-DHA) in both the maternal and fetal compartments. Compared to non-variants, pregnant women who carried the variant allele tended to have lower RBC-DHA across the duration of the study (6.9% vs. 7.4%; main effect, *p* = 0.08) (Figure 2A) and lower cord RBC-DHA upon delivery (7.6% vs. 8.4%; main effect, *p* = 0.09) (Figure 2B). Carriers (versus non-carriers) of the maternal *PEMT* rs4646343 variant did not exhibit significantly different amounts of maternal plasma DHA (main effect, *p* = 0.54) or cord plasma DHA (main effect: *p* = 0.50). Similarly, carriers (versus non-carriers) of the newborn *PEMT* rs4646343 variant did not exhibit significantly different amounts of cord RBC-DHA (main effect, *p* = 0.82) or cord plasma DHA (main effect, *p* = 0.48).

**Figure 3:**
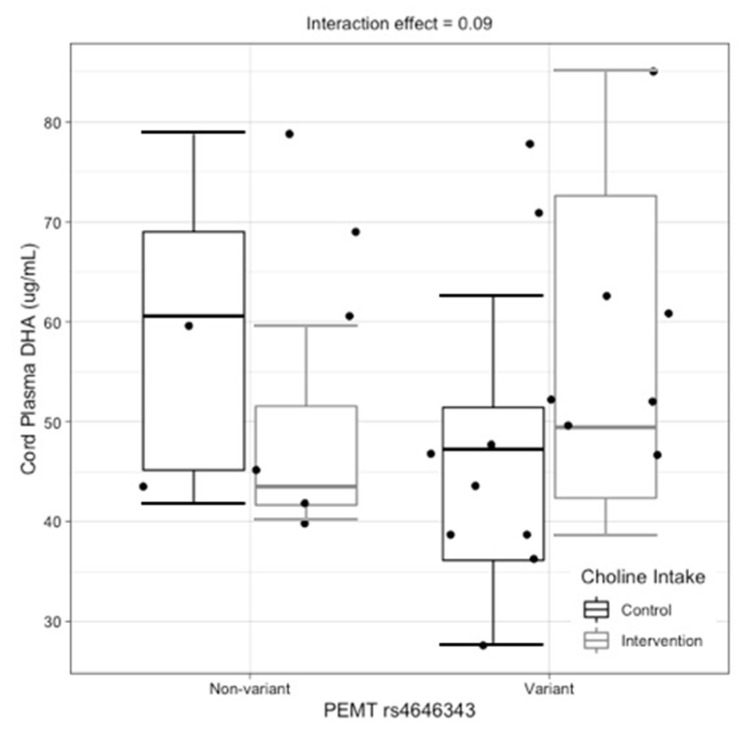
Interactive effects of maternal *PEMT* rs4646343 genotype and maternal choline intake on cord plasma DHA (μg/mL) (raw data boxplot).

The maternal *PEMT* rs4646343 genotype tended to interact with choline intake to influence cord plasma DHA (interaction effect, *p* = 0.09). While no significant differences in cord plasma DHA were detected between intervention arms among non-variant pregnant women (*p* = 0.30), DHA content in cord plasma tended to be higher among variant pregnant women who were supplemented with choline versus variant pregnant women who were not supplemented with choline (*p* = 0.12) (Figure 3).

The maternal or newborn *PEMT* rs4646343 genotype did not significantly interact with time or choline intervention arm for other maternal or cord biomarkers of DHA status (Supplementary Tables S1 and S2).

#### 3.2.2. *MTHFD1* rs2236225

**Figure 4:**
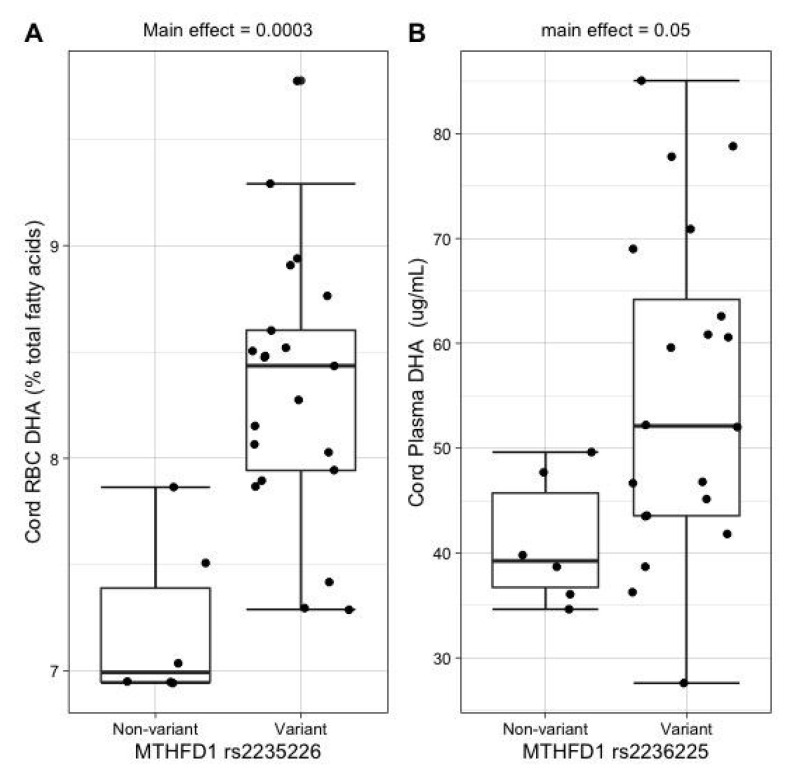
Relationship between maternal *MTHFD1* rs2235226 genotype and cord DHA status indicators (**A**) Cord RBC-DHA (% total fatty acids; raw data boxplot). (**B**) Cord plasma DHA (μg/mL; raw data boxplot).

Carriers (versus non-carriers) of the maternal *MTHFD1* rs2236225 variant exhibited higher DHA in cord RBCs (8.3% vs. 7.3%; main effect, *p* = 0.0003) (Figure 4A) and higher cord plasma DHA (55 vs. 41 μg/mL; main effect, *p* = 0.05) (Figure 4B). Carriers (versus non-carriers) of the maternal *MTHFD1* rs2236225 variant did not exhibit significantly different amounts of maternal RBC-DHA (main effect, *p* = 0.22) or maternal plasma DHA (main effect, *p* = 0.23). Similarly, carriers (versus non-carriers) of the newborn *MTHFD1* rs2236225 variant did not exhibit significantly different amounts of cord RBC-DHA (main effect, *p* = 0.31) or cord plasma DHA (main effect, *p* = 0.55).

The maternal or newborn *MTHFD1* rs2236225 genotype did not significantly interact with time or choline intervention arm for any maternal or cord biomarkers of DHA status (Supplementary Tables S1 and S2).

#### 3.2.3. *PEMT* rs7946

Carriers (versus non-carriers) of the maternal *PEMT* rs7946 variant tended to have higher maternal plasma DHA (main effect, *p* = 0.12). No additional main effects of the maternal or fetal *PEMT* rs7946 genotype were detected on other biomarkers of DHA status in the maternal or fetal compartment (Supplementary Tables S1 and S2).

**Figure 5:**
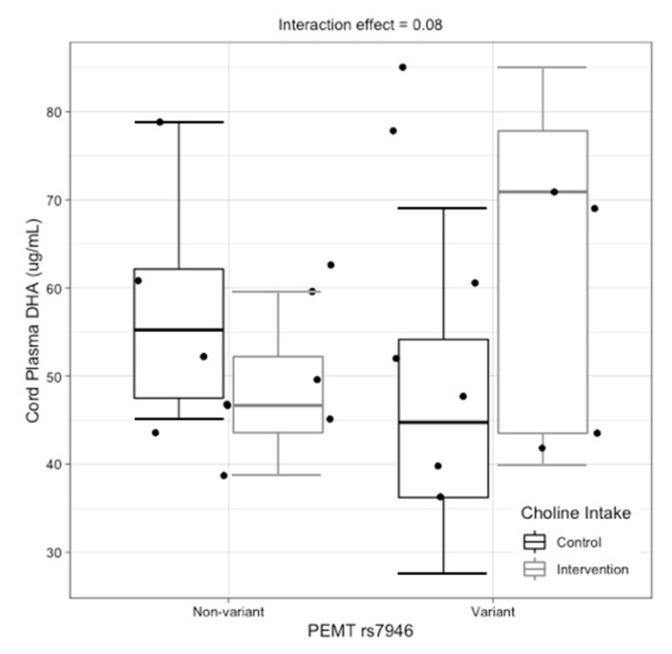
Interactive effects of newborn *PEMT* rs7946 genotype and choline intake on cord plasma-DHA (μg/mL) (raw data boxplot).

The newborn *PEMT* rs7946 genotype tended to interact with choline intake to influence cord plasma DHA (interaction effect, *p* = 0.08). While no significant differences in cord plasma DHA were detected between choline intervention arms among non-variant newborns (*p* = 0.32), DHA content in cord plasma tended to be higher among variant newborns whose mothers were supplemented with choline versus variant newborns whose mothers were not supplemented with choline (*p* = 0.12) (Figure 5).

The maternal *PEMT* rs7946 genotype also tended to interact with choline intake to influence maternal plasma DHA (interaction effect, *p* = 0.10). However, upon stratification by choline intervention arm and further analysis, it became clear that this interaction arose from differences between the intervention arms within the same genotype at baseline. When these baseline differences were controlled for in the statistical analysis, the interaction term became non-significant (main effect of baseline, *p* = 0.003; main effect of interaction, *p* = 0.48).

The maternal or newborn *PEMT* rs7946 genotype did not significantly interact with time or choline intervention arm for other maternal or cord biomarkers of DHA status (Supplementary Tables S1 and S2).

#### 3.2.4. *BHMT* rs3733890

Carriers (versus non-carriers) of the newborn *BHMT* rs3733890 variant exhibited lower cord RBC-DHA (7.7% vs. 8.4%; main effect, *p* = 0.01). No additional main effects of the maternal or newborn *BHMT* rs3733890 genotype on other biomarkers or DHA status were detected.

The maternal *BHMT* rs3733890 genotype interacted with choline intake to influence maternal plasma DHA (interaction effect, *p* = 0.05). However, upon stratification by intervention arm and further analysis, it became clear that this interaction arose from differences between the choline intervention arms within the same genotype at baseline. When these baseline differences were controlled for in the statistical analysis, the interaction term became non-significant (main effect of baseline, *p* = 0.0007; main effect of interaction, *p* = 0.15).

The maternal or newborn *BHMT* rs3733890 genotype did not significantly interact with time or choline intervention arm for other maternal or cord biomarkers of DHA status (Supplementary Tables S1 and S2).

#### 3.2.5. *MTHFR* rs11081133

The maternal or newborn *MTHFR* rs11081133 genotype was not significantly associated with any of the maternal and cord measures of DHA status; model outputs are shown in Supplementary Tables S1 and S2. Trends toward a significant interaction of *MTHFR* rs110811 genotype and time were observed for maternal RBC-DHA (*p* = 0.14).

## 4. Discussion

This post-hoc analysis, conducted in pregnant women and their newborns, sought to investigate the effects of five common variants found in one-carbon metabolizing genes on biomarkers of DHA status. Two main findings emerged: (i) carriers of the maternal *PEMT* rs4646343 variant exhibited borderline lower DHA in maternal RBC membranes across gestation, and (ii) carriers of the maternal *MTHFD1* rs2236225 variant exhibited higher DHA in cord plasma and cord RBC at delivery. 

PEMT performs three sequential methylation reactions to convert phosphatidylethanolamine into phosphatidylcholine (PEMT-PC), using S-adenosylmethionine as a methyl donor [12]. The PEMT pathway has been shown to produce DHA-enriched PC, making genetic variants in the PEMT pathway putative modifiers of DHA status [6,13,14]. The promoter region of the *PEMT* gene has several estrogen-response elements (EREs) that allow for upregulation during pregnancy [15]. The *PEMT* rs4646343 variant has been found to be in high linkage disequilibrium with a second, functional SNP, *PEMT* rs12325817. *PEMT* rs12325817 is proximal to one of these EREs and has been associated with a disrupted ability of *PEMT* to respond to estrogen regulation [16]. In the current study, carriers of the *PEMT* rs4646343 variant tended to have lower DHA in maternal RBCs throughout gestation (6.9% vs. 7.4%; *p* = 0.08) and lower cord RBC-DHA upon delivery (7.6% vs. 8.4%; *p* = 0.09), which is consistent with impaired upregulation of the *PEMT* gene. This could indicate that carriers of this variant require higher DHA intakes in order to achieve a maternal DHA status that is similar to their non-carrier counterparts. 

MTHFD1 is a tri-functional enzyme that catalyzes the interconversions of tetrahydrofolate (THF), 10-formyl-THF, 5,10-methenyl-THF, and 5,10-methylene-THF (Figure 1) [12]. Overwhelmingly, this series of reactions occurs in the order outlined above. The *MTHFD1* rs2236225 variant resides in the 10-formyl-THF synthetase domain, and while this polymorphism does not affect the enzyme’s ability to perform its synthetase activity, it has been found to decrease its half-life and stability [17]. In the present study, carriers of the maternal *MTHFD1* rs2235226 variant exhibited higher cord RBC-DHA (8.3% vs. 7.3%; *p* = 0.0003) and higher DHA in cord plasma (55 vs. 41 μg/mL; *p* = 0.05). This finding was unexpected since a decreased half-life should diminish 5,10-methylene-THF formation, ultimately reducing the supply of methyl groups for the PEMT pathway (Figure 1). Furthermore, previous studies in mice have shown that heterozygosity for a copy of MTHFD1 with no synthetase activity, effectively blocking the MTHFD1 pathway, has deleterious consequences on one-carbon metabolism, and results in lower concentrations of methionine in plasma [18]. One possible explanation for our unexpected finding is that a different enzyme compensates for these impairments in one-carbon metabolism. Serine hydroxymethyltransferase 1 (SHMT1) bypasses the MTHFD1 pathway by using serine to convert THF into 5,10-methylene-THF [12], and mice with a compromised MTHFD1 pathway have lower concentrations of serine in their plasma, suggesting an upregulation of SHMT1 [18]. Thus, it is possible that SHMT1 becomes upregulated to rescue one-carbon metabolism in carriers of the *MTHFD1* 2235226 variant, thereby supporting PEMT activity (Figure 1). Nonetheless, this hypothesis is not fully satisfactory since increases in DHA were not observed in the maternal compartment, and *PEMT* is not expressed in placenta or fetal liver. Compensatory mechanisms at the level of the placenta should be investigated, including fatty acid transport and endogenous synthesis of DHA.

Two interactions between genotype and maternal choline intake were detected, suggesting that choline intake during pregnancy may influence the impact of select one-carbon metabolic genes. Carriers of the variant allele for the maternal *PEMT* rs4646343 genotype and newborn *PEMT* rs7946 genotype tended to exhibit higher DHA content in cord plasma amongst mothers consuming the higher (versus lower) choline intake level. While it is possible that a higher maternal choline intake rescues the effects of these variants, further analysis with larger cohorts is needed to confirm these findings. Indeed, the main limitation of this study is its small sample size, which can yield spurious results. 

To conclude, two common genetic variants, namely *PEMT* rs4646343 and *MTHFD1* rs2236225, were found to be associated with biomarkers of DHA status in this pregnancy cohort. These findings highlight the potential importance of considering variants in one-carbon metabolizing genes when assessing DHA status or determining DHA requirement, particularly at the level of the individual. Such assessments may be especially important among pregnant mothers with other risk factors for low DHA status such as obesity [19,20]. Further research in larger cohorts assessing the relationship of these variants to DHA status and related outcomes (e.g., cognition, pre-term birth), as well as whether DHA supplementation can attenuate the effects of the *PEMT* rs4646343 variant, is warranted.

## Figures and Tables

**Table 1:** Frequency of maternal (*n* = 30) and newborn (*n* = 28) genotypes by choline intervention arm ^1^.

Genotype	Control	Intervention
*PEMT* rs4646343 (Maternal)		
Non-variant	5	4
Variant	10	11
*PEMT* rs4646343 (Newborn)		
Non-variant	6	5
Variant	9	8
*PEMT* rs7946 (Maternal)		
Non-variant	5	8
Variant	10	7
*PEMT* rs7946 (Newborn) ^2^		
Non-variant	6	6
Variant	8	7
*BHMT* rs3733890 (Maternal)		
Non-variant	7	9
Variant	8	6
*BHMT* rs3733890 (Newborn)		
Non-variant	8	8
Variant	7	5
*MTHFD1* rs2236225 (Maternal)		
Non-variant	4	3
Variant	11	12
*MTHFD1* rs2236225 (Newborn)		
Non-variant	7	4
Variant	8	9
*MTHFR* rs11081133 (Maternal)		
Non-variant	9	9
Variant	6	6
*MTHFR* rs11081133 (Newborn) ^2^		
Non-variant	8	5
Variant	7	7

^1^ No differences (*p* > 0.05) were detected in genotype frequency between the control and intervention groups. ^2^ Lower sample number in genotype is due to missing data from one newborn.

**Table 2:** Maternal clinical and demographic characteristics by maternal genotype ^1^.

	*PEMT* rs4646343		*PEMT* rs7946		*BHMT* rs3733890		*MTHFD1* rs2236225		*MTHFR* rs11081133	
	Non- Variant (*n* = 9)	Variant (*n* = 21)	Non- Variant (*n* = 13)	Variant (*n* = 17)	Non- Variant (*n* = 7)	Variant (*n* = 23)	Non- Variant (*n* = 13)	Variant (*n* = 17)	Non- Variant (*n* = 7)	Variant (*n* = 23)
Intervention										
*Control*	5	10	5	10	4	11	5	10	4	11
*Intervention*	4	11	8	7	3	12	8	7	3	12
Age, y [mean (SD)]	31.6 (2.9)	31.9 (5.2)	33.9 (3.0)	33.9 (3.0)	31.4 (4.0)	31.8 (4.0)	31.0 (4.3)	32.3 (3.6)	31.4 (4.0)	31.8 (4.0)
Maternal Race										
*White*	8	20	12	16	7	21	12	16	7	21
*Non-White*	1	1	1	1	0	2	1	1	0	2
Maternal Ethnicity										
*Non-Hispanic*	7	21	12	16	6	22	12	16	6	22
*Hispanic*	2	0	1	1	1	1	1	1	1	1
Pre-Pregnancy BMI [mean (SD)]	23.6 (3.7)	23.9 (3.4)	23.3 (2.7)	23.3 (2.7)	23.9 (2.7)	23.6 (3.8)	23.2 (3.2)	24.1 (3.7)	23.9 (2.7)	23.6 (3.8)
Gestational Age, week [mean (SD)]	13.1 (2.5)	13.8 (1.9)	14.1 (1.8)	14.1 (1.8)	13.6 (1.4)	13.3 (2.5)	12.5 (2.8)	14.0 (1.6)	13.6 (1.4)	13.3 (2.5)
Baseline RBC-DHA, % fatty acids [mean (SD)]	5.9 (0.9)	6.2 (1.0)	6.1 (1.1)	6.1 (1.1)	5.6 (1.1)	6.1 (0.8)	5.9 (0.7)	6.1 (1.0)	5.6 (1.1)	6.1 (0.8)
Baseline plasma DHA, μg/mL [mean (SD)]	88 (20)	90 (30)	91 (33)	91 (33)	78 (9)	92 (26)	82 (13)	94 (29)	78 (9)	92 (26)

^1^ No differences (*p* > 0.05) were detected in genotype frequency between the control and intervention groups.

## Data Availability

Data described in the manuscript, code book, and analytic code will be made available upon request pending request review and approval. Data sharing is dependent upon the nature of request and its compliance with approved data uses by the Institutional Review Board and associated informed consent.

## Supplementary Materials

The following supporting information can be downloaded at: https://www.mdpi.com/article/10.3390/nu14183801/s1, Table S1: P-values for regression models by maternal genotype and outcome variable. For genotype, table shows non-variant vs variant; and for intervention, table shows control vs. intervention. Table S2: P-values for regression models by newborn genotype and outcome variable. For genotype, table shows non-variant vs variant; and for intervention, table shows control vs. intervention. Table S3: DHA biomarker values for all regression models by genotype and outcome variable. The values are estimated marginal means derived from the model ± confidence interval.
