# Supplementary material for: Genetic Variants in One-Carbon Metabolism and Their Effects on DHA Biomarkers in Pregnant Women: A Post-Hoc Analysis
Source: Nutrients. 2022 Sep 15;14(18):3801. doi: 10.3390/nu14183801 (PMC9506554; doi:10.3390/nu14183801)
Supplement: Supplementary file 1 [file nutrients-14-03801-s001.zip › nutrients-1871471-supplementary.pdf]

Supplementary materials

Supplementary Table S1: P-values for regression models by maternal genotype and outcome variable. For genotype, table shows non-variant vs variant; and for intervention, table shows control vs. intervention.

	***PEMT* rs4646343**	***PEMT* *rs7946***	***BHMT* rs3733890**	***MTHFD1* rs2236225**	***MTHFR* rs11081133**
	p-value	*p-value*	*p-value*	*p-value*	*p-value*
**Maternal RBC-DHA, %**					
Genotype	0.08	0.58	0.58	0.22	0.82
Visit	<2e-16	<2e-16	<2e-16	<2e-16	<2e-16
Visit 2					
Visit 3					
Visit 4					
Intervention	0.01	0.04	0.05	0.02	0.04
Genotype x Time	0.7	0.86	0.27	0.72	0.14
Visit 2					
Visit 3					
Visit 4					
Genotype x Intervention	0.31	0.31	0.22	0.24	0.88
**Maternal Plasma-DHA, µg/mL**					
Genotype	0.54	0.12	0.16	0.23	0.80
Visit	1.2e-12	3.1e-13	3.5e-13	4.7e-10	2.0e-12
Visit 2					
Visit 3					
Visit 4					
Intervention	0.01	0.008	0.02	0.03	0.02
Genotype x Time	0.25	0.39	0.27	0.93	0.93
Visit 2					
Visit 3					
Visit 4					
Genotype x Intervention	0.69	0.10	0.05	0.84	0.63
**Cord RBC-DHA, %**					
Genotype	0.09	0.62	0.97	0.0003	0.38
Intervention	0.12	0.12	0.14	0.17	0.15
Genotype x Intervention	0.16	0.32	0.21	0.38	0.86

**Cord Plasma-DHA, μg/mL**					
Genotype	0.50	0.64	0.96	0.05	0.39
Intervention	0.45	0.47	0.51	0.60	0.46
Genotype x Intervention	0.09	0.95	0.56	0.60	0.41

Supplementary Table S2: P-values for regression models by newborn genotype and outcome variable. For genotype, table shows non-variant vs variant; and for intervention, table shows control vs. intervention.

	***PEMT* rs4646343**	***PEMT* rs7946**	***BHMT rs3733890***	***MTHFD1 rs2236225***	***MTHFR rs11081133***
	p-value	*p-value*	*p-value*	*p-value*	*p-value*
**Cord RBC-DHA, %**					
Genotype	0.82	0.62	0.01	0.31	0.90
Intervention	0.16	0.23	0.14	0.22	0.14
Genotype x Intervention	0.74	0.36	0.77	0.78	0.83
**Cord Plasma-DHA, μg/mL**					
Genotype	0.48	0.84	1.00	0.55	0.48
Intervention	0.49	0.60	0.51	0.59	0.49
Genotype x Intervention	0.81	0.08	0.69	0.22	0.81

Supplementary Table S3: DHA biomarker values for all regression models by genotype and outcome variable. The values are estimated marginal means derived from the model ± confidence interval.

**Outcome**	***PEMT*** **rs4646343**		***PEMT*** **rs7946**		***BHMT*** **rs3733890**		***MTHFD1*** **rs2236225**		***MTHFR*** **rs11081133**	
	Non-variant (n = 18)	Variant (n = 12)	Non-variant (n = 9)	Variant (n = 9)	Non-variant (n = 7)	Variant (n = 23)	Non-variant (n = 13)	Variant (n = 17)	Non-variant (n = 7)	Variant (n = 23)
**Maternal genotype**										
Maternal RBC DHA, %										
Visit 1	6.2$^{\pm0.69}$	5.9$^{\pm0.45}$	5.9$^{\pm0.60}$	6.1$^{\pm0.53}$	5.9$^{\pm0.53}$	6.1$^{\pm0.57}$	5.7$^{\pm0.79}$	6.1$^{\pm0.44}$	5.9$^{\pm0.51}$	6.2$^{\pm0.63}$
Visit 2	7.4$^{\pm0.69}$	6.9$^{\pm0.45}$	7.0$^{\pm0.60}$	7.1$^{\pm0.52}$	7.1$^{\pm0.53}$	7.0$^{\pm0.57}$	6.9$^{\pm0.80}$	7.1$^{\pm0.44}$	7.1$^{\pm0.51}$	7.0$^{\pm0.63}$
Visit 3	7.9$^{\pm0.69}$	7.5$^{\pm0.45}$	7.5$^{\pm0.61}$	7.6$^{\pm0.52}$	7.7$^{\pm0.53}$	7.4$^{\pm0.57}$	7.2$^{\pm0.79}$	7.7$^{\pm0.44}$	7.6$^{\pm0.51}$	7.5$^{\pm0.63}$
Delivery	7.9$^{\pm0.69}$	7.4$^{\pm0.45}$	7.6$^{\pm0.61}$	7.6$^{\pm0.52}$	7.7$^{\pm0.53}$	7.4$^{\pm0.57}$	7.4$^{\pm0.80}$	7.6$^{\pm0.43}$	7.7$^{\pm0.51}$	7.4$^{\pm0.63}$
Overall	7.4$^{\pm0.40}$	6.9$^{\pm0.27}$	7.0$^{\pm0.35}$	7.1$^{\pm0.31}$	7.1$^{\pm0.33}$	7.0$^{\pm0.34}$	6.8$^{\pm0.46}$	7.1$^{\pm0.25}$	7.1$^{\pm0.31}$	7.0$^{\pm0.38}$
Maternal Plasma DHA, μg/mL										
Visit 1	93$^{\pm26.6}$	87$^{\pm17.4}$	81$^{\pm21.2}$	97$^{\pm18.3}$	88$^{\pm18.7}$	87$^{\pm19.9}$	80$^{\pm30.1}$	92$^{\pm16.4}$	88$^{\pm18.9}$	90$^{\pm23.2}$
Visit 2	126$^{\pm26.6}$	122$^{\pm17.3}$	113$^{\pm21.1}$	133$^{\pm18.3}$	127$^{\pm18.7}$	117$^{\pm20.0}$	117$^{\pm30.0}$	125$^{\pm16.4}$	125$^{\pm18.9}$	121$^{\pm23.1}$
Visit 3	144$^{\pm26.6}$	127$^{\pm17.4}$	127$^{\pm21.2}$	138$^{\pm18.3}$	139$^{\pm18.7}$	121$^{\pm19.9}$	119$^{\pm30.0}$	136$^{\pm16.5}$	134$^{\pm18.9}$	129$^{\pm23.2}$
Delivery	109$^{\pm26.6}$	113$^{\pm17.4}$	110$^{\pm21.1}$	114$^{\pm18.3}$	117$^{\pm18.7}$	102$^{\pm20.0}$	103$^{\pm30.1}$	114$^{\pm16.5}$	112$^{\pm19.0}$	110$^{\pm23.1}$
Overall	118$^{\pm16.0}$	112$^{\pm10.0}$	108$^{\pm12.2}$	121$^{\pm10.0}$	118$^{\pm11.0}$	107$^{\pm10.9}$	105$^{\pm11.8}$	117$^{\pm9}$	115$^{\pm11.6}$	112$^{\pm14}$
Cord RBC-DHA (%)	8.4$^{\pm0.59}$	7.6$^{\pm0.38}$	8.0$^{\pm0.53}$	8.2$^{\pm0.45}$	8.1$^{\pm0.46}$	8.1$^{\pm0.50}$	7.3$^{\pm0.60}$	8.3$^{\pm0.30}$	8.2$^{\pm0.45}$	8.0$^{\pm0.54}$
Cord Plasma DHA, μg/mL	53$^{\pm13.0}$	51$^{\pm8.5}$	50$^{\pm12.0}$	54$^{\pm9.8}$	52$^{\pm10.5}$	52$^{\pm10.9}$	41$^{\pm15.4}$	55$^{\pm7.9}$	54$^{\pm9.8}$	59$^{\pm11.2}$
Control	59$^{\pm15.9}$	45$^{\pm11.2}$								
Intervention	48$^{\pm20.4}$	57$^{\pm12.5}$								
**Newborn genotype**										
Cord RBC-DHA, %	8.1$^{\pm0.58}$	8.1$^{\pm0.44}$	8.2$^{\pm0.51}$	8.1$^{\pm0.48}$	8.4$^{\pm0.42}$	7.7$^{\pm0.47}$	8.0$^{\pm0.61}$	8.2$^{\pm0.44}$	8.1$^{\pm0.53}$	8.1$^{\pm0.51}$
Cord Plasma DHA, μg/mL	55$^{\pm12.1}$	50$^{\pm9.5}$	53$^{\pm10.8}$	53$^{\pm9.6}$	52$^{\pm10.3}$	52$^{\pm11.1}$	47$^{\pm14.8}$	53$^{\pm9.0}$	56$^{\pm11.5}$	50$^{\pm10.5}$
